# Carotenoid and Tocopherol Profiling in 18 Korean Traditional Green Leafy Vegetables by LC-SIM-MS

**DOI:** 10.3390/foods12061312

**Published:** 2023-03-19

**Authors:** Eun-Young Ko, Ji-Ho Lee, Iyyakkannu Sivanesan, Mi-Jung Choi, Young-Soo Keum, Ramesh Kumar Saini

**Affiliations:** 1 Department of Food Science and Biotechnology of Animal Resources, Konkuk University, Seoul 05029, Republic of Korea; choimj@konkuk.ac.kr; 2 Department of Crop Science, Konkuk University, Seoul 05029, Republic of Korea; micai1@naver.com (J.-H.L.); rational@konkuk.ac.kr (Y.-S.K.); saini1997@konkuk.ac.kr (R.K.S.); 3 Department of Bioresources and Food Science, Institute of Natural Science and Agriculture, Konkuk University, Seoul 05029, Republic of Korea; siva74@konkuk.ac.kr

**Keywords:** *Pimpinella brachycarpa*, *Taraxacum mongolicum*, Toona sinensis, phytochemicals, provitamin A, lutein, β-carotene

## Abstract

Fruits and vegetables are a vital source of redox-active phytochemicals in the diet. Traditional green leafy vegetables (GLVs) are a rich source of carotenoids, dietary fiber, minerals, phenols, vitamins, and tocopherols and are commonly consumed in rural areas worldwide. In traditional Korean medicine, many GLVs are used to treat various ailments. However, data on the carotenoid and tocopherol content of many traditional GLVs consumed in the Republic of Korea are insufficient. The current work aims to compare the carotenoid and tocopherol profiles of 18 traditional GLVs by utilizing a single ion monitoring LC-MS approach to identify the potential GLVs for commercial cultivation and healthy diet formulations. Among the traditional GLVs investigated, (all-E)-lutein was the most abundant carotenoid, ranging from 44.4% in *Glehnia littoralis* to 52.1% in *Heracleum moellendorffii*. It was followed by (all-E)-violaxanthin and (all-E)-β-carotene. The highest contents of (all-*E*)-violaxanthin (75.6 µg/g FW), 9-Z-neoxanthin (48.4 µg/g FW), (all-*E*)-luteoxanthin (10.8 µg/g FW), (all-*E*)-lutein (174.1 µg/g FW), total xanthophylls (310.5 µg/g FW), (all-*E*)-β-carotene (69.6 µg/g FW), and total carotenoids (380.1 µg/g FW) were recorded in *Pimpinella brachycarpa*. Surprisingly, *Taraxacum mongolicum* also showed the highest contents of (all-*E*)-violaxanthin, (all-*E*)-lutein, and total carotenoids, which were statistically non-significant (*p* > 0.05, Tukey HSD) with *P. brachycarpa*. The highest concentration of (all-*E*)-zeaxanthin (14.4 µg/g FW) was recorded in *Solidago virga-aurea*. Among the studied herbs, 13.9 (*H. moellendorffii*)–133.6 µg/g FW (*Toona sinensis*) of α-tocopherol was recorded. Overall, the results suggest that *P. brachycarpa* and *T. mongolicum* are rich sources of carotenoids. On the other hand, *T. sinensis* is a rich source of α-tocopherol. These GLVs can be utilized in the diet to enhance the intake of health-beneficial carotenoids and α-tocopherol.

## 1. Introduction

The World Health Organization recommends adequate intake (400–500 g per day) of fruits and vegetables (including green leafy and cruciferous vegetables) to minimize the risk of high blood pressure, coronary heart disease, and stroke [1]. Green leafy vegetables, or GLVs, are an important part of a healthy diet as they are rich in essential nutrients and phytochemicals with health benefits. These include dietary fiber, vitamins, minerals, carotenoids, and polyphenolic compounds.

Clinical trials have also demonstrated the advantages of the enhanced intake of vegetables and fruits in reducing the risk of developing chronic and metabolic disorders, including cancer, type 2 diabetes, obesity, and cardiovascular and neurological diseases [2,3,4]. The redox-active phytochemicals involving carotenoids and tocopherols in fruits and vegetables help prevent these disorders by minimizing free radical-mediated oxidative damage to proteins, cellular lipids, DNA, and other protein biomolecules [5,6,7,8].

Vitamin E, also known as tocols, which includes four tocotrienols (α-, β-, γ- and δ) and four tocopherols (α-, β-, γ- and δ), differs by the position of methyl groups on the chromanol ring [9]. Tocols serve as critical components of cellular lipids. They neutralize free radicals, thus preventing the free radical-mediated oxidative damage of lipids and minimizing the incidence of diseases associated with oxidative stress [10,11,12,13].

Carotenoids are mainly tetraterpenoid (C40) pigments commonly synthesized de-novo by photoautotrophs, including higher plants. Animals rely on provitamin A carotenoids (converted by the body into vitamin A, e.g., β-cryptoxanthin and α- and β-carotene) as a dietary source to carry out vital functions. Additionally, carotenoids without pro-vitamin A activity (e.g., xanthophylls) have antioxidant abilities that shield against chronic and metabolic ailments, as well as photooxidative harm to the skin and eyes in animals [7,14].

The Republic of Korea is well known for its traditional high-vegetable diet, which is probably responsible for the significantly lower rates of chronic diseases than other industrialized countries with similar economic development [1]. Several traditional GLVs such as *Amaranthus lividus* L., *Angelica gigas* Nakai, *Glehnia littoralis* F. Schmidt ex Miq., *Heracleum moellendorffii* Hance, *Peucedanum japonicum* Thunb., *Pimpinella brachycarpa* (Kom.) Nakai, *Aralia continentalis* Kitag., *Kalopanax septemlobus* (Thunb. ex A.Murr.) Koidz., *Artemisia princeps* Pamp., *Cirsium setidens* Nakai, *Ligularia fischeri* (Ledeb.) Turcz., *Petasites japonicus* (Siebold & Zucc.) Maxim., *Rudbeckia laciniata* L., *Solidago virga-aurea* L. var. *asictica* Nakai, *Taraxacum mongolicum* Hand.-Mazz., *Adenophora triphylla* (Thunb.) A.DC. var. *japonica* (Regel) H. Hara, *Allium victorialis* var. *platyphyllum* Makino, and *Toona sinensis* (A.Juss.) M.Roem. are sold at local markets in Korea (Table 1). The extracts and compounds obtained from traditional GLVs (1–18) have been shown to possess antioxidant [15,16,17,18,19,20,21,22], anticancer [18,23,24,25,26], antiinflammation [20,25,27,28], anti-melanogenic [29,30], anti-fatigue [31], anti-obesity [32,33], antidiabetic [34], and immunostimulatory [35] activities. GLVs have abundant phytopigments. Several studies have confirmed the content of α-carotene, β-carotene, lutein, violaxanthin, zeaxanthin, and α-tocopherol in a few traditional GLVs [15,16,36,37,38,39,40]. However, data on carotenoid content and compositions of several traditional GLVs consumed in Korea are still unavailable. Moreover, GLVs are not widely investigated for tocopherol content. Thus, quantifying bioactive phytochemicals in these species can help identify potential GLVs for healthy food formulations.

A report by Yoon et al. [10] revealed that GLVs consumed in the Republic of Korea are good sources of carotenoids (β-carotene and lutein), and their contents are higher than other commonly consumed plant foods. The authors [10] investigated the contents of β-carotene, lutein, and total phenolic in several vegetables consumed in Korea.

Given the information presented above, this study aimed to determine the levels and composition of carotenoids and tocols (Vitamin E) in 18 different types of traditional GLVs using liquid chromatography (LC)–mass spectrometry (MS) with a single ion monitoring (SIM) approach.

## 2. Materials and Methods

### 2.1. Reagents, Standards, and Plant Materials

An authentic standard of tocols mix (α-, β-, γ-, and δ-tocotrienols and α-, β-, γ-, and δ-tocopherols) was obtained from ChromaDex (ChromaDex Inc., Irvine, CA, USA). (all-*E*)-β-carotene was procured from Merck Ltd., Seoul, Republic of Korea. (all-*E*)-lutein, 9-*Z*-neoxanthin, (all-*E*)-violaxanthin used in this investigation were isolated from lettuce, while (all-E)-zeaxanthin was prepared from corn seeds using our established protocol [41]. An acid-catalyzed reaction was used to transform (all-*E*)-luteoxanthin from (all-*E*)-violaxanthin [42].

The solvents used in the study were of LC grade and sourced from J.T. Baker^®^ located in Suwon-Si, Republic of Korea.

The 18 traditional green leafy vegetables were collected from natural habitats and the traditional market, as detailed in Table 1. The vegetables were brought to the lab, cleaned, individually packed in Ziplock polythene bags, and stored at −90 °C in an ultra-low temperature deep freezer (CLN-2300CW, Nihon Freezer Co., Ltd., Yushima, Japan) until analysis.

### 2.2. Extraction of Carotenoids and Tocols

The lipophilic bioactive carotenoids and tocols were simultaneously extracted from fresh foliage using our recently optimized method [43]. In sum, a 2 g fresh sample was placed into a Falcon 50 mL conical centrifuge tube and homogenized with 25 mL of a solvent mixture (acetone/ethanol/cyclohexane, 1:1:2, *v*/*v*) containing 0.1% butylated hydroxytoluene (BHT) as an antioxidant [44]. The mixture was then subjected to bath sonication (JAC-2010; 300 w, 60 Hz, for 10 min) and ultra-shaking for 2 min in collomix viba x.30 (Tinting Solutions B.V., Nederland) to ensure complete extraction. The sample was vacuum filtered and pellets were extracted again until obtaining the colorless pellets. The filtrate containing lipophilic compounds were pooled, transferred to a 300 mL Short Neck Boiling flask (round bottom), and dried in a vacuum rotary evaporator at 35 °C. The extract containing carotenoids and other lipophilic compounds were recovered in 4 mL of acetone containing 0.1% BHT and transferred to a 5 mL glass vial fitted vial with a PTFE-lined screw cap closure. A small portion of the extract was filtered using a Nylon syringe filter (pore size 0.45 μm; Whatman) and transferred to an amber HPLC vial for the analysis of tocols and carotenoids.

The carotenoids and tocols were analyzed in their non-hydrolyzed form, as the hydrolysis process can lead to the degradation of these compounds [45].

### 2.3. LC-MS Analysis

To analyze the tocols and carotenoids, a liquid chromatography (LC)–mass spectrometry (MS) with a single ion monitoring (SIM) approach was employed. The LC-MS/SIM analysis was carried out using an LCMS-9030 quadrupole time-of-flight (Q-TOF) mass spectrometer manufactured by Shimadzu in Tokyo, Japan. The analysis was performed in an atmospheric pressure chemical ionization (APCI; Positive mode), following the LC separation in a YMC C30 carotenoid column (150 mm × 4.6 mm, 3 μm; YMC, Wilmington, NC) maintained at 20 °C. The solvent system was methanol/water (95:5; *v*/*v*) containing 5 mM of ammonium formate (Mobile Phase A) and methyl tertiary butyl ether/methanol/water (90:7:3, *v*/*v*/*v*) containing 5 mM of ammonium formate (Mobile Phase B). Ammonium formate was added as an ionization enhancer in the mass spectrometer. The gradient elution program involved starting at 0% B at 0 min and reaching 100% B at 45 min, followed by a 5 min post-run at 0% B. The flow rate was maintained at 0.5 mL/min. The source and compound parameters were optimized as follows: drying gas flow, 10 L/min; nebulizing gas flow, 3 L/min; corona needle voltage, 4.0 kv; interface temperature, 400 °C; DL temperature, 300 °C; heat block temperature, 300 °C; Q1 resolution, ±20 ppm; and data acquisition (sampling), 1.85625 Hz [43]. Quantitative analysis was performed using the selected ion monitoring (SIM) mode. Table 2 lists the optimized SIM transitions (*m*/*z*). To quantify each carotenoid and tocol compound, external standards were used. The linearity range for each standard compound can be found in Table A1.

### 2.4. Calculation of Vitamin A Activity

The vitamin A activity, as retinol activity equivalents (RAEs), was calculated based on the in vivo conversion factor of 1 µg RAE = 12 µg of β-carotene proposed by the Food and Nutrition Board, Institute of Medicine (IOM), USA [46].

### 2.5. Statistical Evaluation and Quality Assurance

Three separate replicates of extraction and analysis were performed for each green leafy vegetable (GLV). The statistical analysis was conducted using IBM SPSS statistics version 25, including a one-way analysis of variance (ANOVA) with a significance level of 0.05 and post hoc testing with Tukey B HSD.

The lower limits for detection (LOD) and quantitation (LOQ) of utilized LC-MS methods were determined based on a signal-to-noise (S/N) ratio of more than 3 and more than 10, respectively [47].

Moreover, the employed LC-MS/SIM method was tested for precision (ability to produce consistent and reproducible results), linearity (relationship between the concentration of the analyte and its response), and accuracy (closeness of the measured value to the true value of the analyte) [48,49].

To calculate the precision of the instrument (both inter-day and intra-day) for chromatographic retention time and peak area measurement, multiple injections of the same concentration within the working range were performed, and the coefficient of variation (% CV) was calculated. The intra-day precision was determined by performing six replicate injections of the same concentration in a single day. On the other hand, to establish the inter-day precision, the standard compounds were analyzed six times over two separate days that were not consecutive.

## 3. Results and Discussion

### 3.1. Validation of LC-MS/SIM Methodology

The LC-MS/SIM method used to quantify carotenoids and tocols underwent validation to assess its accuracy, precision, and linearity [48,49]. The coefficient of variation (CV; a ratio of the standard deviation (SD) to the mean of the peak area counts) or relative standard deviation (RSD) was measured and found to be <0.35% and 9.23% (inter-day and intra-day) for chromatographic retention times and peak area counts, respectively, for carotenoids and tocopherols (Table A1). The calibration curves demonstrated a high coefficient of correlation (r2; >0.999–1.000) between standard concentrations and corresponding peak area counts. These findings provide evidence that the employed LC-MS/SIM method is reliable and can be used with confidence.

### 3.2. Carotenoid Composition

**Figure 1:**
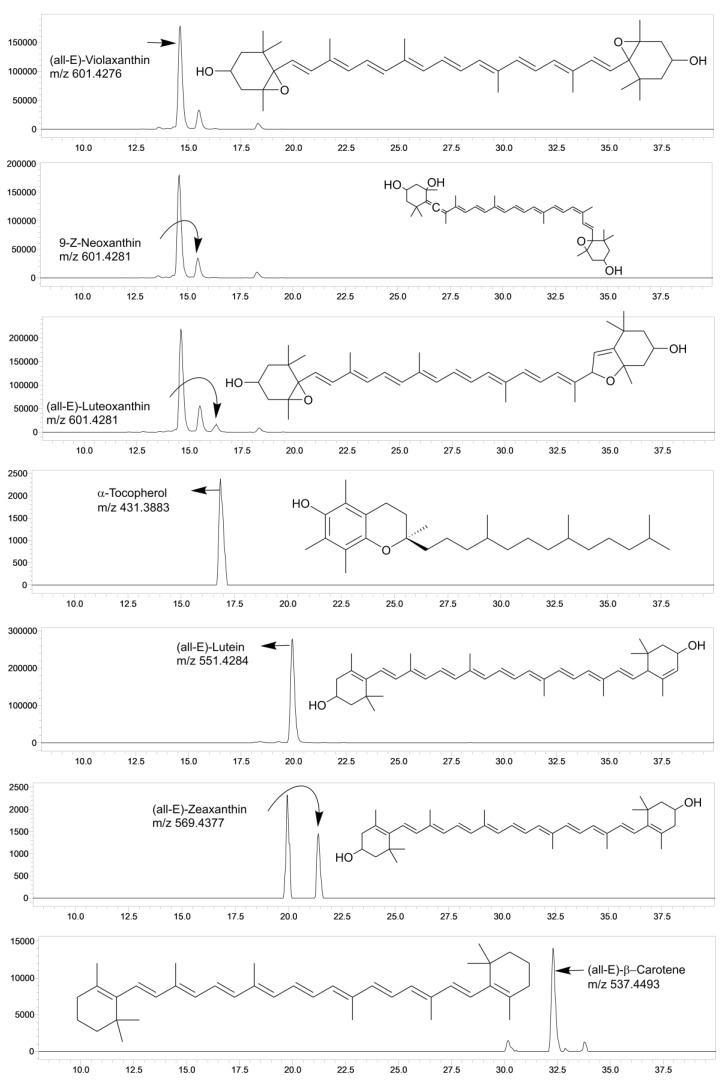
Representative LC-SIM/MS chromatograms of the carotenoids and α-tocopherol detected in the studied traditional green leafy vegetables (GLVs).

Carotenoids are crucial bioactive substances that greatly influence the nutritional quality and appealing color of food [50]. In the present investigation, six major carotenoids, including five xanthophylls ((all-*E*)-zeaxanthin), (all-*E*)-lutein, (all-*E*)-luteoxanthin, 9-Z-neoxanthin, and (all-*E*)-violaxanthin) and a provitamin A carotenoid (all-*E*)-β-carotene were quantified (Figure 1; Table 3 and Table 4). The quantified levels of all identified carotenoids were significantly higher than the limit of quantification (LOQ) (Table A1).

Among the traditional GLVs investigated in the present investigation, the (all-*E*)-lutein (β,ε-carotene-3,3′-diol) was the most prominent carotenoid ranging between 44.4 (*Glehnia littoralis*)–52.1% (*Heracleum moellendorffii*) of total carotenoids, followed by (all-*E*)-violaxanthin (5,6:5′,6′-diepoxy-5,5′,6,6′-tetrahydro-β,β-carotene-3,3′-diol) and (all-*E*)-β-carotene (Table 3 and Table 4). The highest contents (µg/g FW) of (all-*E*)-lutein (174.1), (all-*E*)-luteoxanthin (10.8), 9-Z-neoxanthin (48.4), (all-*E*)-violaxanthin (75.6), total xanthophylls (310.5), (all-*E*)-β-carotene (69.6), and total carotenoids (380.1) were recorded in *Pimpinella brachycarpa*. Surprisingly, *Taraxacum mongolicum* also showed the highest contents of (all-*E*)-violaxanthin, (all-*E*)-lutein, and total carotenoids, which were statistically non-significant with *Pimpinella brachycarpa*. In contrast, *Solidago virga-aurea exhibited* the highest contents (14.4 µg/g FW) of (all-*E*)-zeaxanthin among all of the traditional GLVs investigated.

Only a few GLVs investigated in the present study were previously explored for carotenoid composition and content. Sathasivam et al. [40] also recorded the dominance of lutein and β-carotene in *Heracleum moellendorffii* leaves, with a total carotenoid content of 1668 µg/g dry weight (DW). In *Pimpinella brachycarpa,* Yoon et al. [51] recorded 54.5 and 32.3 µg/g FW of lutein and β-carotene, respectively. In contrast, we recorded 174.1 and 69.9 µg/g FW of lutein and β-carotene, respectively. Similarly, in *Toona sinensis*, 223 µg/g FW of lutein and 186 µg/g FW β-carotene are reported by Cheng et al. [36], which is substantially greater than the contents documented in the present investigation.

Kao et al. [52] recorded the prominence of (all-*E*)-β-carotene, followed by (all-*E*)-violaxanthin, 9-Z-neoxanthin, and (all-*E*)-lutein in *Taraxacum officinale*, a close relative of *T. mongolicum* investigated in the present study. It is commonly known as dandelion and is traditionally used for heat relieving, detoxification, diuretic, and hepatoprotective activities [53,54].

The carotenoid compositions and contents varied significantly among the different plants. Moreover, a significant variation has been documented among the species of the same genus. In a study of the carotenoid composition of medicinally important GLVs consumed in India, a near 3-fold variation was recorded for the total carotenoid content among the leaves of three species of the genus *Amaranthus*, with the highest total carotenoid content in *A. viridis* L. (2538 µg/g DW), followed by *A. gangeticus* L. (789 µg/g DW), and *A. tristis* L. (675 µg/g DW) [39].

The (all-*E*)-β-carotene is the provitamin A carotenoid predominantly found in herbs. The recommended dietary reference intake (DRI) of vitamin A for adult men is 900 retinol activity equivalents (RAEs) according to the dietary guidelines [46]. The vitamin A content calculated as the RAE, using the conversion of 1 RAE = 12 μg of β-carotene, revealed that the consumption of 100 g of herbs investigated in the present study can supply the 24.8 (*Kalopanax septemlobus*)–64.4 % (*Pimpinella brachycarpa*) DRI of vitamin A (Table 4).

Along with the provitamin A carotenoids, the traditional GLVs investigated in the present investigation are found to be rich in (all-*E*)-lutein. Lutein and zeaxanthin are pigments in the macula that act as filters for blue light, thus protecting the retina and maintaining vision [55]. Research has demonstrated that a higher intake of these compounds can support eye health [55]. Thus, among the traditional GLVs studied in the present investigation, *Pimpinella brachycarpa* and *Taraxacum mongolicum* are the richest sources of (all-*E*)-β-lutein; thus, their enhanced intake may help to improve ocular health.

We have previously explored the carotenoid contents of several herbs, including baby leaf vegetables [56], green and green/red perilla (*Perilla frutescens* Britt.) [57], and 23 diverse lettuce cultivars [58]. In baby leaf vegetables, the (all-*E*)-β-carotene content ranged from 19.3 to 60.2 µg/g FW, with the total carotenoid content ranging from 57.1 to 195.2 µg/g FW [56]. In green/red and green perilla foliage, the (all-*E*)-β-carotene content was 51.2–52.1 µg/g FW, with a total carotenoid content of 196.1–209.4 µg/g FW [57]. Among the 23 different lettuce cultivars, the (all-*E*)-β-carotene content w ranged from 4.2 to 13.6 g/g FW, with the total carotenoid content ranging from 54.4 to 129.8 g/g FW [58]. In this investigation, the contents of (all-*E*)-β-carotene were 26.8–69.6 µg/g FW, with a total carotenoid content of 193.3–380.1 µg/g FW, indicating that traditional GLVs investigated in the present study are more concentrated sources of carotenoids than commonly consumed GLVs. These results are also supported by a recent study by Lee et al. [59], who observed much higher contents of β-carotene in underutilized GLVs, such as Moringa foliage (108 µg/g FW), sweet leaf bush (125 µg/g FW), and sweet potato foliage (110 µg/g FW), compared to iceberg lettuce (4 µg/g FW).

### 3.3. Tocols Composition

**Figure 2:**
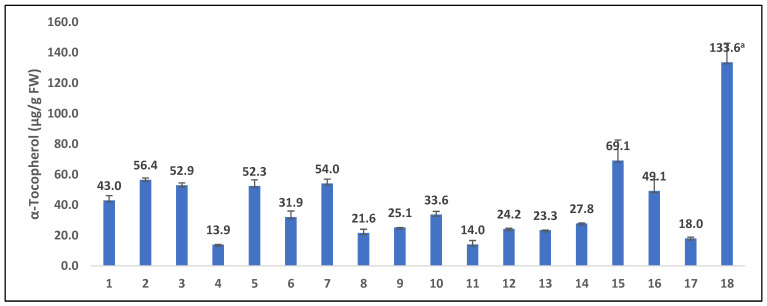
The contents of α-tocopherol in studied traditional GLVs. The results represent the average ± standard deviation (SD) obtained from three replicate analyses. The values with the superscript letter “a” show the highest (*p* < 0.05, Tukey B HSD) contents among the various traditional GLVs. The numbers 1–18 on the *X*-axis represent the S/No. of samples in Table 1.

The term “tocols” encompasses four forms of tocopherols (α-, β-, γ-, and δ-) and four forms of tocotrienols (α-, β-, γ-, and δ-) [60]. In the present study, the tocol content and composition were analyzed using an LC-SIM-MS-based method. Among the studied herbs, 13.9 (*Heracleum moellendorffii*)–133.6 µg/g FW (*Toona sinensis*) of α-tocopherol was recorded, whereas other types of tocopherols and tocotrienols were not detected in a significant amount (Figure 2).

Limited studies exist on the alpha-tocopherol content of green leafy vegetables (GLVs), as most research has been concentrated on seed oil. Previous studies on α-tocopherol levels in GLVs have revealed significant variation. Among the foliage of several edible tropical plants, α-tocopherol contents ranged between 6.9 (*Brassica oleracea*)–426.8 (*Sauropus androgynus*) µg/g FW [61]. Among the several GLVs commonly consumed in Southeast Asia, 1.9 (green amaranth)–183 µg/g FW (foliage of *Moringa oleifera*) of α-tocopherols was documented by Lee et al. [59].

Our recent investigation found that α-tocopherol levels in leaf mustard varied among the four cultivars studied, with recorded amounts ranging from 67.2 (cv. Asia Curled) to 83.4 µg/g FW (cv. Cheong) [62]. In another recent study on GLVs, α-tocopherol levels ranging from 22.0 µg/g FW in spinach to 87.7 µg/g FW in Moringa were recorded [43]. Considering these previous reports, *Toona sinensis* foliage investigated in the present study is a rich source of α-tocopherol.

α-tocopherol plays a key role as a chain-breaking antioxidant, thus preventing the free radical-mediated oxidative damage of lipids and minimizing the incidence of diseases associated with oxidative stress, such as heart disease, certain types of cancer, and age-related cognitive decline [10,11,12,13]. Additionally, α-tocopherol may help improve skin health and immune function [63].

The DRI of α-tocopherol for both women and men is 15.0 mg per day [13]. Among the various forms of tocols, α-tocopherol has the maximum vitamin E activity, with 1 mg equaling 1 α-TE [13]. Vegetable oils, mainly wheat germ oil, are the richest source of tocols in the diet [13]. Nevertheless, taking into account the highest α-tocopherol content (133.6 µg/g FW), *Toona sinensis* foliage can provide 90% of the DRI of vitamin E.

## 4. Conclusions

In this study, 18 traditional green leafy vegetables (GLVs) were analyzed for their carotenoid and tocol content using LC-MS/SIM. Among the studied GLVs, the most abundant carotenoid was (all-*E*)-lutein, followed by (all-*E*)-violaxanthin and (all-*E*)-β-carotene. The highest content of carotenoids was found in *Pimpinella brachycarpa* and *Taraxacum mongolicum*, while the highest content of (all-*E*)-zeaxanthin was recorded in *Solidago virga-aurea*. In contrast, the highest α-tocopherol content was found in *Toona sinensis*. The results suggest that *P. brachycarpa* and *T. mongolicum* are good sources of carotenoids, while *T. sinensis* is a good source of α-tocopherol. Adding these conventional GLVs to the diet can provide an optimal way to obtain the maximum nutritional benefits of α-tocopherol and carotenoids.

## Figures and Tables

**Table 1:** List of different traditional green leafy vegetables (GLVs) investigated in the present study.

S/No.	Family	Scientific Name	Local Name	Place of Collection
1	Amaranthaceae	*Amaranthus lividus* L.	Chambireum	a
2	Apiaceae	*Angelica gigas* Nakai	Chamdangwi	a
3		*Glehnia littoralis* F. Schmidt ex Miq.	Haedangpung	a
4		*Heracleum moellendorffii* Hance	Uhsuri	a
5		*Peucedanum japonicum* Thunb.	Gatgireum	a
6		*Pimpinella brachycarpa* (Kom.) Nakai	Chamnamul	a
7	Araliaceae	*Aralia continentalis* Kitag.	Ttangdureup	a
8		*Kalopanax septemlobus* (Thunb. ex A.Murr.) Koidz.	Eomnamu	c
9	Asteraceae	*Artemisia princeps* Pamp.	Suk	a
10		*Cirsium setidens* Nakai	Gondre	b
11		*Ligularia fischeri* (Ledeb.) Turcz.	Gomchwi	a
12		*Petasites japonicus* (Siebold & Zucc.) Maxim.	Mowi	b
13		*Rudbeckia laciniata* L.	Samipgukhwa	b
14		*Solidago virga-aurea* L. var. *asictica* Nakai	Miyeokchwi	a
15		*Taraxacum mongolicum* Hand.-Mazz.	Mindle	a
16	Campanulaceae	*Adenophora triphylla* (Thunb.) A.DC. var. *japonica* (Regel) H. Hara	Jandae	a
17	Liliaceae	*Allium victorialis* var. *platyphyllum* Makino	Sanmaneul	d
18	Meliaceae	*Toona sinensis* (A.Juss.) M.Roem.	Chamjuknamu	a

a. Purchased from the Hanaro market, Seoul, Republic of Korea, in April 2022. b. Collected from the farm grown in Gangwon Province, Republic of Korea, in May 2022. c. Collected from Hadenter Farm, Gyeongbuk Province, Republic of Korea, in May 2022. d. Collected from Ulleung Agricultural Technology Center grown in Gyeongbuk, Republic of Korea, in May 2022.

**Table 2:** The selected ion monitoring (SIM) transitions (*m/z*) utilized for carotenoid and tocol analysis.

Class of Compounds	Compound	Transition (*m/z*) *
Carotenoids	(all-*E*)-β-carotene; β,β-carotene	537.4493
	(all-*E*)-zeaxanthin; β,β-carotene-3,3′-diol	569.4377
	(all-*E*)-lutein; β,ε-Carotene-3,3′-diol	551.4284
	(all-*E*)-luteoxanthin; β-carotene-3,3′-diol, 5,6:5′,8′-diepoxy-5,5′,6,8′-tetrahydro-	601.4281
	9-*Z*-neoxanthin; 5′,6′-epoxy-6,7-didehydro-5,6,5′,6′-tetrahydro-β,β-carotene-3,5,3′-triol	601.4281
	(all-*E*)-violaxanthin; 5,6,5′,6′-diepoxy-5,6,5′,6′-tetrahydro-β,β-carotene-3,3′-diol)	601.4276
Tocopherols 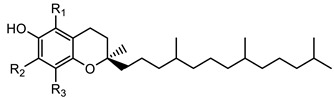	α- tocopherol (R_1_ = R_2_ = R_3_ = CH_3_)	431.3883
	β-tocopherol (R_1_ = R_3_ = CH_3_; R_2_ = H)	416.3669
	γ-tocopherol (R_1_ = H; R_2_ = R_3_ = CH_3_)	416.3669
	δ-tocopherol (R_1_ = R_2_ = H; R_3_ = CH3)	402.3488
Tocotrienols 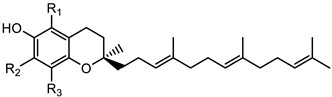	α- tocotrienol (R_1_ = R_2_ = R_3_ = CH_3_)	425.3423
	β-tocotrienol (R_1_ = R_3_ = CH_3_; R_2_ = H)	411.3268
	γ-tocotrienol (R_1_ = H; R_2_ = R_3_ = CH_3_)	411.3268
	δ-tocotrienol (R_1_ = R_2_ = H; R3 = CH3)	397.3113

* The selection of the transition (*m*/*z*) was based on the protonated precursor ion that was most noticeable among the standard compounds.

**Table 3:** The xanthophyll contents (µg/g FW) in the studied traditional GLVs.

S/No.	(all-*E*)-Violaxanthin	9-*Z*-Neoxanthin	(all-*E*)-Luteoxanthin	(all-*E*)-Lutein	(all-*E*)-Zeaxanthin	Total Xanthophylls
1	47.7 ± 2.11	27.0 ± 3.38	1.17 ± 0.11	113.5 ± 5.08	0.49 ± 0.01	189.9 ± 10.4
2	64.3 ± 5.30	29.0 ± 1.51	0.74 ± 0.08	138.7 ± 5.78	1.25 ± 0.22	233.9 ± 12.89
3	67.2 ± 1.11	30.1 ± 0.79	2.66 ± 0.54	130.9 ± 0.63	2.27 ± 0.50	233.0 ± 1.23
4	35.8 ± 4.4	22.1 ± 1.75	2.10 ± 0.56	111.6 ± 6.0	0.35 ± 0.03	171.9 ± 12.7
5	43.9 ± 0.35	23.2 ± 0.62	2.53 ± 0.86	100.3 ± 0.73	0.39 ± 0.05	170.3 ± 2.61
6	75.6 ± 5.95 ^a^	48.4 ± 2.37 ^a^	10.8 ± 1.14 ^a^	174.1 ± 6.51 ^a^	1.64 ± 0.12	310.5 ± 13.57 ^a^
7	43.9 ± 4.33	16.5 ± 2.14	1.85 ± 0.20	96.5 ± 3.34	1.02 ± 0.07	159.8 ± 10.1
8	54.2 ± 5.64	17.3 ± 2.58	1.33 ± 0.03	97.9 ± 3.83	0.39 ± 0.12	171.1 ± 12.2
9	66.3 ± 1.05	31.6 ± 1.47	1.89 ± 0.34	128.6 ± 4.38	1.85 ± 0.13	230.3 ± 6.43
10	49.2 ± 4.41	23.2 ± 0.61	3.74 ± 1.64	112.2 ± 5.76	2.17 ± 0.35	190.5 ± 12.1
11	67.2 ± 6.14	29.6 ± 2.43	1.31 ± 0.17	130.0 ± 9.38	1.12 ± 0.06	229.3 ± 17.9
12	48.8 ± 0.99	24.5 ± 0.49	2.624 ± 1.17	110.8 ± 0.50	1.02 ± 0.28	187.8 ± 1.45
13	70.4 ± 2.04	27.8 ± 2.27	1.68 ± 0.19	139.3 ± 10.5	1.76 ± 0.10	240.9 ± 14.7
14	61.8 ± 4.02	21.5 ± 2.17	1.23 ± 0.07	120.6 ± 4.54	14.44 ± 0.07 ^a^	219.6 ± 10.9
15	75.1 ± 2.44 ^a^	41.2 ± 0.94	2.04 ± 0.31	163.7 ± 4.95 ^a^	0.94 ± 0.28	283.0 ± 7.05
16	75.3 ± 1.47 ^a^	22.9 ± 1.37	1.19 ± 1.18	126.4 ± 3.81	3.51 ± 0.13	229.3 ± 7.70
17	49.0 ± 7.07	22.5 ± 1.13	1.39 ± 0.49	100.5 ± 4.77	0.63 ± 0.18	174.1 ± 12.7
18	59.1 ± 0.77	18.1 ± 0.09	7.97 ± 3.21	107.9 ± 5.95	2.53 ± 0.21	195.6 ± 10.2

The results represent the average ± standard deviation (SD) obtained from three replicate analyses. The values with the superscript letter “a” show the highest (*p* < 0.05, Turkey B HSD) contents among the various traditional GLVs. The S/No. 1–18 represents the S/No. of samples in Table 1.

**Table 4:** The (all-*E*)-β-carotene, total carotenoids, and retinol activity equivalents (RAEs) in studied traditional GLVs.

S/No.	(all-*E*)-β-Carotene *	Total Carotenoids (TC) *	% (all-*E*)-Lutein in TC	% (all-*E*)-β-Carotene in TC	RAE (µg)	% DRI from 100 g **
1	57.9 ± 4.75	247.8 ± 15.2	45.8 ± 0.76	23.4 ± 0.49 ^a^	4.83 ± 0.40	53.6 ± 4.40
2	46.0 ± 0.16	280.0 ± 13.0	49.5 ± 0.24	16.5 ± 0.71	3.83 ± 0.01	42.6 ± 0.14
3	61.6 ± 0.46	294.6 ± 1.69	44.4 ± 0.47	20.9 ± 0.04	5.13 ± 0.04	57.0 ± 0.43
4	42.5 ± 4.6	214.5 ± 17.2	52.1 ± 1.41 ^a^	19.8 ± 0.55	3.55 ± 0.38	39.4 ± 4.25
5	37.2 ± 0.44	207.5 ± 3.04	48.4 ± 0.36	17.9 ± 0.05	3.10 ± 0.04	34.4 ± 0.40
6	69.6 ± 3.92 ^a^	380.1 ± 17.5 ^a^	45.8 ± 0.40	18.3 ± 0.19	5.80 ± 0.33 ^a^	64.4 ± 3.63 ^a^
7	33.5 ± 0.47	193.3 ± 10.5	50.0 ± 1.00	17.4 ± 0.71	2.80 ± 0.04	31.1 ± 0.43
8	26.8 ± 1.86	197.9 ± 14.05	49.5 ± 1.58	13.5 ± 0.02	2.23 ± 0.15	24.8 ± 1.72
9	53.6 ± 0.53	283.9 ± 5.90	45.3 ± 0.60	18.9 ± 0.58	4.47 ± 0.04	49.7 ± 0.49
10	39.1 ± 3.68	229.6 ± 15.7	48.9 ± 0.85	17.0 ± 0.44	3.26 ± 0.31	36.2 ± 3.41
11	47.7 ± 1.98	277.0 ± 19.8	46.9 ± 0.03	17.3 ± 0.52	3.98 ± 0.17	44.2 ± 1.84
12	46.7 ± 1.94	234.5 ± 0.49	47.3 ± 0.12	19.9 ± 0.79	3.890 ± 0.16	43.2 ± 1.80
13	59.3 ± 2.58	300.2 ± 17.3	46.4 ± 0.83	19.8 ± 0.28	4.94 ± 0.22	54.9 ± 2.39
14	42.1 ± 3.17	261.7 ± 14.0	46.1 ± 0.74	16.1 ± 0.35	3.51 ± 0.26	39.0 ± 2.93
15	65.4 ± 1.27	348.4 ± 8.31 ^a^	47.0 ± 0.30	18.8 ± 0.08	5.45 ± 0.11	60.5 ± 1.17
16	46.3 ± 1.29	275.6 ± 8.99	45.9 ± 0.11	16.8 ± 0.08	3.86 ± 0.11	42.9 ± 1.19
17	43.0 ± 0.69	217.0 ± 13.3	46.3 ± 0.65	19.8 ± 0.90	3.58 ± 0.06	39.8 ± 0.64
18	31.1 ± 3.83	226.7 ± 14.1	47.6 ± 0.33	13.7 ± 0.84	2.59 ± 0.32	28.8 ± 3.55

The results represent the average ± standard deviation (SD) obtained from three replicate analyses. The values with the superscript letter “a” show the highest (*p* < 0.05, Tukey B HSD) contents among various traditional GLVs. The S/No. 1–18 represents the S/No. of samples in the table. * The contents are expressed as µg/g FW. ** Considering the recommended dietary reference intake (DRI) of 900 μg RAE/day for adults.

## Data Availability

The data are contained within the article.

## Appendix A

**Table A1:** The validation parameters for the liquid chromatography–mass spectrometry with a single ion monitoring (LC-MS/SIM) analysis of carotenoids and tocopherols.

Compounds	Working Range (μg/mL)	Area Counts Precision		Retention Time Precision		Limits		Correlation Coefficient (R^2^)
Compounds		Intra-Day CV (%, *n* = 6)	Inter-Day CV (%, *n* = 6 × 2)	Intra-Day CV (%, *n* = 6)	Inter-Day CV (%, *n* = 6 × 2)	LOQ (μg/g)	LOD (μg/g)	
(all-*E*)-violaxanthin	5–50	4.11	5.30	0.07	0.15	0.32	0.11	0.999
9-Z-neoxanthin	5–50	7.01	7.42	0.09	0.17	0.84	0.28	0.999
(all-*E*)-lactucaxanthin	5–50	4.68	5.43	0.08	0.18	0.87	0.29	1.000
(all-*E*)-lutein	5–50	2.53	4.98	0.07	0.16	0.15	0.05	0.999
(all-*E*)-zeaxanthin	5–50	5.12	7.06	0.09	0.14	0.46	0.15	1.000
(all-*E*)-β-carotene	5–50	4.91	6.05	0.08	0.17	0.68	0.23	1.000
α-tocopherol	10–100	8.82	9.23	0.23	0.35	2.94	0.97	0.999

LOQ, limits of quantitation; LOD, limits of detection; CV, coefficient of variation.
